# Perinephric Hematoma and Hemorrhagic Shock as a Rare Presentation for an Acutely Obstructive Ureteral Stone with Forniceal Rupture: A Case Report

**DOI:** 10.1089/cren.2016.0033

**Published:** 2016-04-01

**Authors:** Firas G. Petros, Debra L. Zynger, Geoffrey N. Box, Ketul K. Shah

**Affiliations:** ^1^ Department of Urology, The Ohio State University Wexner Medical Center, Columbus, Ohio.; ^2^ Department of Pathology, The Ohio State University Wexner Medical Center, Columbus, Ohio.

## Abstract

***Background:*** Spontaneous perinephric hematoma (SPH) secondary to a forniceal rupture as the first presenting sign for an obstructive ureteral stone in a patient without history of urolithiasis has not been described previously.

***Case presentation:*** We report a 70-year-old Caucasian male patient who presented to our emergency room with fever, altered mental status, and left flank pain. He had a temperature of 103.3°F, tachycardia, but stable blood pressure. He had left flank tenderness. A computed tomography scan of the abdomen/pelvis with intravenous contrast revealed an intracapsular hematoma (13.3 × 10.0 × 6.4 cm) with an active bleeding and a 1.1 cm left proximal ureteral stone. The patient became quickly hemodynamically unstable and was taken for emergent exploratory laparotomy and left nephrectomy. An active bleeding was encountered secondary to a (2.4 × 2.0 cm) lateral capsular defect in the kidney.

***Conclusion:*** Hemorrhagic/septic shock as a presenting sign for an obstructive ureteral stone may require an emergent nephrectomy in a hemodynamically unstable patient.

## Introduction

Acute ureteral obstruction secondary to ureteral stones in septic patients is considered a life-threatening event requiring immediate decompression of the obstructed kidney and treatment with empiric antibiotic therapy.^1^ There are well-recognized risks from delaying drainage of an obstructed and infected system, including morbidity from sepsis, acute renal failure, and even mortality.^2^ Renal forniceal rupture with urinary extravasation into the retroperitoneum is an uncommon complication of acute ureteral obstruction. However, the majority of reported cases are caused by ureteral calculi.^3^ Subscapular hematoma in association with urolithiasis has been described after extracorporeal shock wave lithotripsy^4^ and ureteroscopic lithotripsy.^5^ However, spontaneous perinephric hematoma (SPH) secondary to a forniceal rupture as the first presenting sign for an obstructive ureteral stone in a patient with no prior history of urolithiasis has not been described previously.

## Case Presentation

**FIG. 1.**
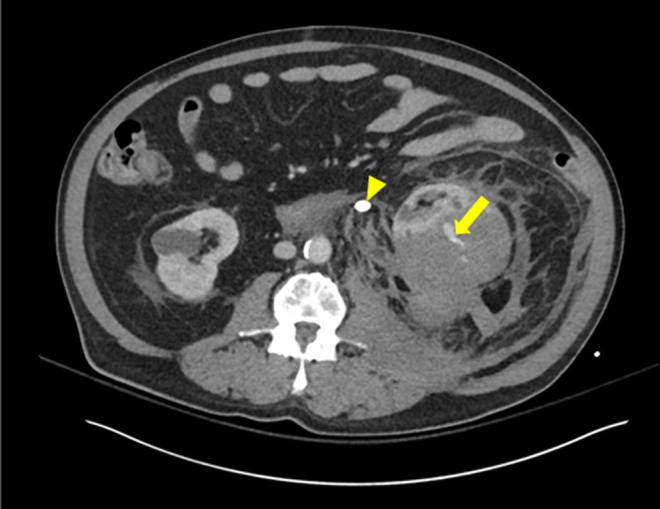
CT scan of abdomen and pelvis with IV contrast axial section showing perinephric hematoma, which deformed and compressed the kidney anteriorly. *Arrow* points to an area of active extravasation of contrast within the hematoma. *Arrow head* points to 1.1 cm obstructive proximal ureteral stone. Marked left perinephric stranding was noted. CT, computed tomography.

**FIG. 2.**
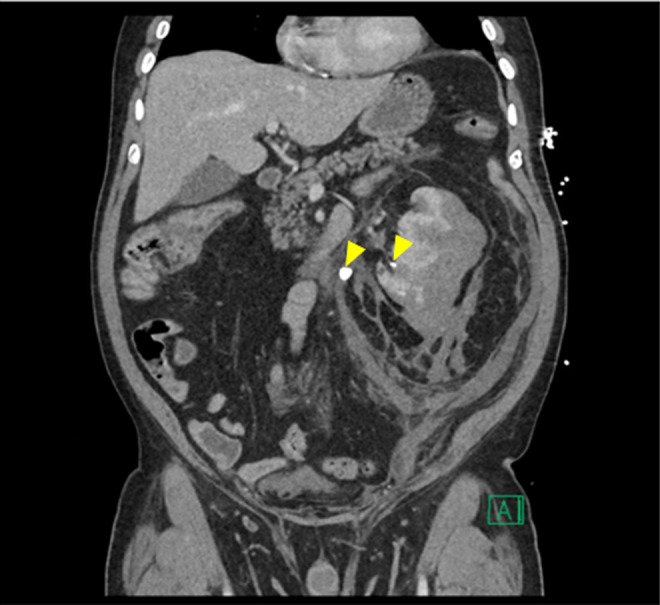
CT scan of abdomen and pelvis with IV contrast coronal section showing perinephric hematoma, nonobtructive lower pole 6 mm stone and 1.1 cm obstructive proximal ureteral stone (*arrow heads*). Marked left perinephric stranding with extension of extracapsular hematoma to distal aorta, iliac vessels, and presacral space was noted.

We report a 70-year-old male patient who presented to our emergency room with fever and altered mental status for 24 hours before presentation. The patient was also complaining of increasing left flank pain, nausea, and emesis. There was no history of trauma. On physical examination, the patient appeared to be in distress and minimally responsive. Initial vital signs revealed a temperature of 103.3°F, tachycardia, but stable blood pressure. Abdominal examination revealed distended abdomen and left flank tenderness to palpation. He was found to have severe leukopenia with white blood cell count of 0.7 K/μL and a serum creatinine (sCr) of 1.64 mg/dL with a normal coagulation profile. A computed tomography (CT) scan of the abdomen/pelvis with intravenous contrast showed a large intracapsular hematoma (13.3 × 10.0 cm), which deformed and compressed the kidney anteriorly (Figs. 1 and 2). There were multiple areas of curvilinear hyperdense material within the hematoma, indicating vascular extravasation consistent with active bleeding. Marked left perinephric stranding with extension of the extracapsular hematoma to the distal aorta, iliac vessels, presacral space, and along the left prerenal space was seen (Fig. 2). A 1.1 cm stone was identified within the proximal left ureter, and a 6 mm stone was identified within the inferior pole of the left kidney. The proximal left ureter was dilated (Figs. 1 and 2).

**FIG. 3.**
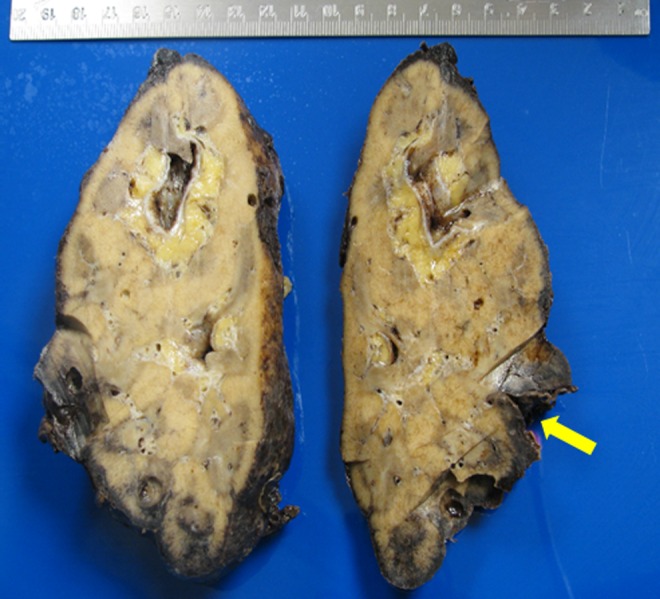
Gross photograph of the bivalved left kidney. *Arrow* points to interpolar area of parenchymal rupture at the lateral aspect of the kidney.

**FIG. 4.**
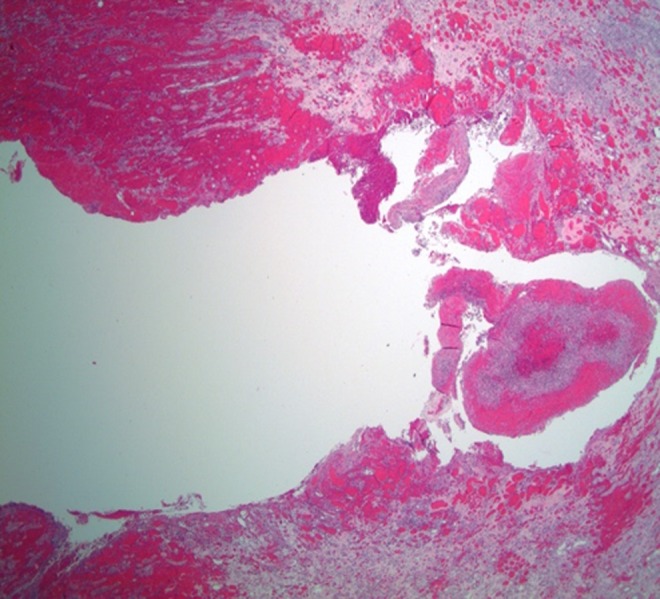
Rupture of renal cortex with acute hemorrhage within and surrounding the defect.

Despite ongoing resuscitative measures with intravenous fluid boluses and intravenous antibiotics, the patient became hemodynamically unstable 1 hour after presentation to the emergency room. His blood pressure decreased to 70/40 mmHg and Hgb dropped to 9.5 g/dL (from 11.3) that required initiation of blood transfusion and infusion of vasoactive pressor medications. The patient remained critically ill, tachycardic, and hypotensive with a blood pressure of 80/60 mmHg despite 8 L of intravenous crystalloid fluid, 4 U of blood, and pressure support. Interventional radiology was consulted. If managed nonoperatively, it appeared the patient would need both a nephrostomy tube and selective arterial embolization. Given the large hematoma with minimal hydronephrosis combined with the patient's rapid clinical deterioration, interventional radiology did not think they would be able to help with both of these problems. Because of the complicated nature of the combined issues of active renal hemorrhage with hemorrhagic/septic shock, the patient was subsequently taken for emergent exploratory laparotomy and left nephrectomy. During the exploration, active bleeding was encountered with a (2.4 × 2.0 cm) shaggy defect at the interpolar lateral aspect of the kidney (Fig. 3). Vascular control of the renal pedicle was obtained and a nephrectomy was performed. Postoperatively, the patient made a steady progress until he was discharged with sCr 1.38 mg/dL, estimated glomerular filtration rate 51 mL/min/1.73 sqM, and stable Hgb 9.6 g/dL. The initial blood and urine cultures revealed growth of *Escherichia coli* that was treated with appropriate antibiotics. Histopathologic examination of the kidney showed acute and chronic interstitial and intratubular inflammatory infiltrate with acute hemorrhage with no evidence of malignancy (Fig. 4).

## Discussion

Urinary extravasation secondary to forniceal rupture has been reported to result from obstruction by ureteral calculi with the majority being within distal ureter.^3^ Although it has been described as a renoprotective phenomenon,^6^ the increase in caliceal pressure that exceeds the tensile strength of forniceal tissues results in tissue disruption and extravasation of urine and possibly blood when involving adjacent parenchymal blood vessels.

SPH is a rare entity, even more rare when the etiology is nephrolithiasis. SPH should always be attributed to underlying pathologic abnormalities of the kidney because of the high incidence of associated renal tumors. Spontaneous rupture of renal cell carcinoma was first described in the 1950s.^7^ Malignant tumors are responsible for SPH in almost one-third of the cases. Approximately half of all cases with spontaneous renal hemorrhage are due to renal tumors, of which angiomyolipoma is the second most common.^8^ Infectious etiology has only rarely been described in association with a perinephric hematoma. Known history of nephrolithiasis in association with infection has been described in a case report as a rare cause of spontaneous rupture of the kidney.^9^

We describe a unique case in a patient with no prior diagnosis of nephrolithiasis who presented with hemorrhagic and septic shock secondary to an acutely obstructive ureteral calculus, resulting in a large perinephric hematoma. The patient's initial clinical constellation of fever, hemodynamic instability, and confusion were likely explained by his perinephric hematoma secondary to active bleeding identified on CT scan in the setting of urinary obstruction with underlying infection. However, the fact that the patient did not respond appropriately to resuscitative measures and blood transfusion mandated an aggressive management course. The patient rapidly improved clinically after a life-saving nephrectomy.

CT scan remains the investigation of choice for diagnosing perinephric/subcapsular hematomas and may be able to delineate the potential underlying etiology. Nonoperative interventions with selective angiography and embolization of the offending arterial bleeder are often the treatment of choice if other surgery is not indicted.^10^ In a hemodynamically unstable patient presenting with acute renal forniceal rupture and perinephric hematoma caused by acutely obstructive ureteral stone similar to the case presented, a decision must be made quickly. When possible, percutaneous management to control the bleeding and to drain the kidney is likely ideal as this would potentially salvage the kidney. Alternatively, a ureteral stent combined with angioembolization would also be an option. The case presented represents a very unique situation where these options seemed unlikely to work in a very unstable patient, so the decision was made to proceed with surgical exploration, acknowledging when done in this setting will most often result in a nephrectomy.

Management of a hemodynamically unstable patient with sepsis from an obstructive stone is emergent decompression of the kidney. Management of a hemodynamically unstable patient with renal trauma is emergent surgical exploration (or angioembolization in selected cases). This case had the combination of both of these issues. Given the location of the perinephric hematoma and the minimal degree of hydronephrosis, interventional radiology did not feel they would be able to place both a nephrostomy tube and embolize the kidney. Furthermore, the outcome of renal embolization in the presence of an active infection is unknown and concerns for being able to clear the infection with devitalized tissue did exist. Minimally invasive nephron-sparing options at this point would be embolization with a ureteral stent, and while this option was entertained, ultimately the decision was made to proceed with surgical exploration as the most definitive and life-saving management option for both of the patient's problems given his ongoing significant hemodynamic instability, concern for progression to death, and uncertainty regarding serious complications if emergent surgical exploration was not performed. This case was approached similar to a renal trauma, through a midline transabdominal approach, allowing for the option of obtaining early vascular control before opening the retroperitoneum. Given the concomitant infection, there was significant tissue inflammation making the dissection more difficult, requiring en bloc stapling across the renal hilum rather than individual division of the renal artery and vein.

## Conclusion

Renal forniceal rupture and perinephric hematoma may result from acute obstruction caused by ureteral calculi. Hemorrhagic/septic shock as a presenting sign for an obstructive ureteral stone requiring an emergent nephrectomy in a hemodynamically unstable patient who failed resuscitative measures has not been described previously.

## Abbreviations Used

CT: computed tomography
sCr: serum creatinine
SPH: spontaneous perinephric hematoma
